# Malgaigne on Pseudo-Strangulation, or Simple Inflammation of Hernia

**Published:** 1842-06

**Authors:** 


MALGAIGNE *on Pseudo-Strangulation, or Simple Inflammation of Hernia.*—Strangulation is said to exist whenever a hernia cannot be reduced, and when pain, constipation, vomiting, and hiccup are present. Should these symptoms progress rapidly, the strangulation is called inflammatory; if slowly, the strangulation is said to be from obstruction (*par engouement*). In either case, an operation is judged indispensable, so soon as the symptoms have acquired a certain degree of intensity. Boyer says, that we should operate if the symptoms continue to increase; and, in old or weakly subjects, he advises us to operate within the first three or four days.

Here we may ask, did Boyer reflect on the true danger of the operation, when he laid down such rules? Assuredly he did; but, with Pott, he thought "that the operation for strangulated hernia presented, of itself, no danger." In order to determine how far this opinion was founded on fact, I have noted all the operations performed during five years (1836–41) in all the Parisian hospitals, and I find that in 183 cases there occurred no less than 114 deaths. Again, in old people, (from 50 to 80,) on whom Boyer would have us to operate so promptly, I find that 70 out of 97 died. Obstruction, Boyer informs us, is produced by a collection of hardened fæces in the hernial tumor, and this frequently occurs in old hernia. But, we may ask, is this latter a fact? For my own part, I may say, that I have examined more than 3000 cases of hernia, without observing the circumstance on which Boyer insists. At Bicêtre, I have examined two of the most voluminous herniæ on record; one tumor measured 10⅓ inches in height, and 22 inches in circumference; the other, 8 inches in height, by 23½ in circumference; but in neither was there any trace of fæcal matter. Besides, we can have hard or fæcal matter in the large intestine alone, and the presence of the latter in a hernial sac is not one-twentieth as frequent as that of the small intestine. Hence, in 95 out of 100 cases, this obstruction, such as it is described in books, is materially impossible; it can only occur when the hernia is formed by the large intestine. As regards the latter case, I can affirm that I have never seen such accumulation of fæcal matter in any autopsy that I have made; and, having consulted authors, I find but a single example in which this collection had really taken place.

The cause, then, of this species of strangulation is imaginary; so, I regret to say, are the symptoms which we find detailed in surgical writings. I have myself been misled by the doctrines laid down. I once operated on a case of supposed strangulated hernia from obstruction; there was no obstruction, no strangulation, and my patient died. Similar errors of this kind have been committed by Pott, Dupuytren, and Sir

A. Cooper; and were all the cases published, we should have a long list of them. But is there any way of avoiding such errors? It seems to me, that some few rules of a simple nature may be established.

1. There is simple, non-inflammatory strangulation, which produces gangrene in a few hours.

2. We have simple inflammation, which is of frequent occurrence, and generally limited to the serous lining of the hernia.

3. Finally, inflammation of the whole mass of the tumor; this latter circumstance, however, is generally a consequence of one of the former conditions, and may be excited by the stricture, or by frequent attempts to effect reduction.

Obstruction of the intestine is a mere creation of the fancy; it is inflammation of the serous lining of the hernial tumor which has been mistaken for it. This peritonitis is one of the most frequent complications of hernia, and surgeons have probably overlooked it from not having sufficiently studied hernial tumors in their simple state. It may exist under the forms of adhesive or suppurative inflammation; the adhesive is often slight, and indicated by passing colic, or even by symptoms of indigestion, which sometimes go so far as vomiting and hiccup; the tumor is now irreducible, and the taxis will only aggravate all the symptoms; but rest in the horizontal position, and cold topical applications, will often suffice to effect reduction; in more severe cases, it may require several days or weeks before the inflammation is sufficiently reduced to allow of the return of the intestine. When hernial tumors, in this state, are reduced, nothing generally remains, except some thickening and irregularity of surface at the lower part of the sac; should the tumor not be reduced, then adhesions take place.

The suppurative inflammation is infinitely more rare, and can scarcely be discovered, except after operation or death of the patient. How are we to distinguish this inflammation of the peritoneal membrane from true strangulation, and what should our practice be? The following are the results of my experience on these points.

1. In no case of old and voluminous hernia, where bandages have not been employed, or have been left off for a considerable time, do we find real strangulation; the openings are much larger than the neck of the hernial tumor, and strangulation cannot occur. This is the result of all my observations on the living and dead body.

2. In cases of simple epiplocele, inflammation of the peritoneal membrane is what is commonly taken for strangulation; I say *commonly*, for I would not deny that strangulation may occur; I have never seen a case, nor have I found an authentic one in surgical works.

3. Hence, in the two cases just mentioned, the operation is contra-indicated; the taxis may be tried at the origin or decline of the inflammation, and the treatment should be purely antiphlogistic.—*Am. Jour. Med. Sci.*, from *Prov. Med. and Surg. Journ.*, Oct. 2, 1841.

